# Anti-inflammatory effects of minocycline are mediated by retinoid signaling

**DOI:** 10.1186/s12868-018-0460-x

**Published:** 2018-09-21

**Authors:** Vera Clemens, Francesca Regen, Nathalie Le Bret, Isabella Heuser, Julian Hellmann-Regen

**Affiliations:** Section Clinical Neurobiology, Department of Psychiatry and Psychotherapy, Campus Benjamin Franklin, Charité - University Medicine Berlin, Hindenburgdamm 30, 12203 Berlin, Germany

**Keywords:** Minocycline, Retinoic acid, Neuroinflammation, Microglia, Cytokines

## Abstract

**Background:**

Minocycline is a lipophilic tetracycline of increasing appeal in neuroscience as it inhibits microglial activation, a mechanism involved in numerous neuropsychiatric disorders. Own data point towards retinoid-mediated effects of minocycline in murine brain and skin, and towards a vicious cycle of neuroinflammation which is driven by microglial activation-induced breakdown of local retinoids such as retinoic acid (RA). We therefore sought to study minocycline’s anti-inflammatory effects on human microglial-like monocyte-derived cells in the context of retinoid signaling.

**Results:**

As hypothesized, minocycline exposure resulted in a substantial increase of RA levels in the human monocytic cell line THP-1. While pro-inflammatory stimulation with lipopolysaccharides resulted in increased tryptophane-degrading indoleamine-2,3-dioxygenase IDO-expression and TNF-α levels in primary human monocyte-derived microglial-like cells, this effect was attenuated by minocycline only in the presence of retinoids. The anti-inflammatory effects of minocycline on TNF-α expression were completely abolished by a pharmacological blockage of retinoic acid receptors (RARs) using BMS-493 and unaffected by selectively blocking retinoid-X-receptors using UVI-3003.

**Conclusions:**

Our data indicate for the first time a RA-dependent, anti-inflammatory effect for minocycline in human microglial-like cells via inhibition of local RA turnover. The RA-dependent mode of action for minocycline appears to be predominantly mediated through RAR-signaling.

## Background

Minocycline is a well-established lipophilic tetracycline and has recently gained much attention in the neuroscience context due to its potent ability to block microglial activation [1–6], a mechanism involved in numerous neuropsychiatric disorders [7] including major depressive disorder [8, 9], schizophrenia [10], autism [11, 12] and Alzheimer’s Disease [13, 14].

Being the main immune cell of the central nervous system, microglia are the central actors in neuroinflammation [7]. By synthesizing pro-inflammatory cytokines like IL-1 β [15], TNF-α or IL-6 [16, 17] as well as superoxide radicals [18], this multi-facetted cell type has been demonstrated to affect neurotransmitter metabolism, various neuroendocrine functions and various aspects of neural plasticity [19]. Under normal conditions, microglia keep brain homeostasis in balance. However, upon sensing pathological stimuli, such as proinflammatory cytokines, alterations in pH or hypoxia, a transformation of the cells, often termed “activation”, occurs that may result in a chronically sustained, vicious circle of “sub-threshold” inflammation. This subtle, yet chronically maintained pro-inflammatory condition is discussed to be the neurobiological correlate underlying the neuropsychiatric phenotype [20]. Furthermore, cytokines stimulate indoleamine-2,3-dioxygenase (IDO), the key enzyme of the kynurenine pathway (KP), that degrades the serotonin precursor tryptophan [21], resulting not only in a decreased serotonin synthesis but also in a microglial-mediated increase of the neurotoxic NMDA-receptor agonist quinolinic acid [22]. The kynurenine pathway represents not only a measure for inflammatory stimulation, but furthermore sets an important bridge between neuro-inflammation and transmitter imbalances that are known for several neuropsychiatric disorders.

For these reasons, microglial activation represents a promising target for the treatment of numerous neuropsychiatric diseases. Preclinical evidence points towards an inhibition of microglial activation by minocycline [1–6], and clinical research indicates efficacy of minocycline in schizophrenia [23–27] and major depression [28–32], disorders where microglial activation is discussed as part of an underlying neuropathology. The mechanisms underlying these promising effects of minocycline, however, remain incompletely understood.

While own previous research points towards effects of minocycline on the homeostasis of endogenous neuroprotective and anti-inflammatory retinoic acid (RA) [33, 34], RA itself is known to inhibit microglial activation [35]. Moreover, in the clinical setting, RA exposure appears to trigger CNS side effects similar to those that are known for minocycline treatment, namely an increased intracranial pressure (pseudotumor cerebri; PTC) [36]. The risk to develop PTC can even be potentiated upon simultaneous exposure to RA and minocycline or other tetracycline derivatives [37]. This association had previously led us to hypothesize an interaction between minocycline and RA at the level of local RA degradation, the crucial step in the regulation of local tissue levels of RA [36–38]. Based on these findings, we hypothesized a potential role of RA signaling in minocycline’s anti-inflammatory actions.

Therefore, we aimed to assess the potential of minocycline in inhibiting microglial activation with and without the presence of retinoids and therefore to further analyze the role of RA signaling in minocycline’s pleiotropic properties.

## Methods

### Materials

All chemicals were purchased from Biochrom (Berlin, Germany), unless otherwise stated.

### THP-1 cell culture

Human THP-1 monocyte cell line (provided by Dr. Ulrike Erben) was cultured in Dulbecco’s Modified Eagle’s Medium (DMEM) containing 10% fetal calf serum (FCS) and 1% penicillin (100 U/mL) and streptomycin (100 μg/mL). Cells were seeded in 96-well plates at an initial density of 8 × 10^4^ per well and allowed to attach for 24 h.

To assess the inhibition of RA-degradation by minocycline, cells were treated with 1 µM all-trans RA (Sigma-Aldrich, St. Louis, USA) and minocycline (Minocyclinhydrochlorid, Hovione, Loures, Portugal) at concentrations of 0, 10 and 100 µM.

To further investigate the effects of inflammatory activation on RA degradation, and to quantify the impact on minocycline on the latter, cells were treated with 100 ng/mL lipopolysaccharide, a pro-inflammatory stimuli (LPS; *Escherichia coli*, Sigma-Aldrich). Vehicle or minocycline were added at 10 and 100 µM. After an incubation period of 24 h, all-trans RA was added to a final concentration of 1 µM.

### Quantification and HPLC analysis of retinoids

For protein denaturation, precipitation and retinoid extraction, 2 vol of acetonitrile (200 µL) were added to each well of the 96-well cell culture plates. Plates were immediately frozen and stored at − 80 °C until further analysis. Within 2 weeks, all samples were further processed by thawing plates on ice, followed by transfer to a microcentrifuge tube and centrifugation at 14,000 rpm, 4 °C, for 15 min. 100 µL of the resulting supernatant were transferred to glass vials and subjected to reversed-phase HPLC analysis (Agilent 1100 model liquid chromatography system equipped with a 1290 Infinity diode array detector; Agilent Technologies, Böblingen, Germany). Analysis of retinoids was performed as previously published [33]. In brief, retinoids and degradation products were separated on a Supelco Suplex column (5 µm, 2.1 × 250 mm; Sigma-Aldrich, Taufkirchen, Germany) using a mobile phase composed of acetonitrile, 2% (w/v) ammonium acetate in water, methanol, glacial acetic acid and n-butanol in a ratio of 69:16:10:3:2 vol. Isocratic elution was performed at a flow rate of 0.65 ml/min and resulted in complete separation of the all-trans RA isomer from 13-, 9-, and 9-, 13-di-cis isomers within a total analysis time of 12 min. All compounds were frequently verified by authentic standards, intra-assay variability, as assessed for the concentration range between 10 and 100 nM was well below 5% CV. Peak purity was monitored by online spectral analysis.

### Primary macrophages

Human peripheral blood mononuclear cells (PBMCs) were obtained from healthy controls and prepared by traditional density gradient cell separation using Histopaque (Sigma-Aldrich) and according to the manufacturer’s instructions. PBMCs were counted and seeded at an initial density of 5 × 10^5^ cells per well.

To generate microglia-like cells, PBMCs were first grown in RPMI-1640 containing 10 µg/mL human cytokine M-CSF (Miltenyi Biotec, Bergisch Gladbach, Germany), 10% FCS, 1% penicillin (100 U/mL) and streptomycin (100 μg/mL) at 37 °C in 5% CO_2_ in a humidified atmosphere. After 7 days, all non-adherent cells were discarded and adherent monocytes were washed in phosphate-buffered saline (PBS) once. Cells were further differentiated in DMEM, also supplemented with 10 µg/mL M-CSF, 1% FCS and 1% penicillin and streptomycin for additional 3 days.

In all subsequent experiments, cell cultures were first treated with a 20-fold stock solution containing either vehicle, all-trans RA, retinol, minocycline or a combination of minocycline plus retinoids at the concentrations resulting in the desired final concentrations of each compound.

Moreover, for all experiments aimed at measuring gene expression changes, cell cultures were supplemented with metabolically competent rat cortex-derived synaptosomes (50 µg/mL). These were added to simulate the metabolic features of a brain-like microenvironment and were prepared from rat brain tissue as previously described [34].

After a 24 h incubation period, pro-inflammatory stimulation was performed by adding LPS to a final concentration of 1 ng/mL. Further analyses were performed 2.5 h after LPS-treatment.

For RA receptor blockage, medium alone, pan-RXR receptor antagonist UVI-3003 (1 µM) or pan-RAR receptor antagonist BMS-493 (1 µM; both from R&D Systems, Minneapolis, USA) were added to the cell culture prior to the addition of minocycline and/or retinoids.

The study was approved by the ethics committee of the Charité—University Medicine Berlin. Written informed consent was obtained from the participants.

### RNA extraction and quantitative PCR

For RNA extraction, TRIzol LS Reagent (Thermo Fisher Scientific, Waltham, USA) was added to the cell culture after medium was removed and cell culture was rinsed twice with PBS. cDNA was synthesized using the RevertAid™ RT Reverse Transcription Kit (Thermo Fisher Scientific) according to manufacturer’s instructions. Polymerase chain reaction (PCR) amplification was performed using a real time PCR cycler and monitoring of SYBR Green I fluorescence (Thermo Fisher Scientific). Relative gene expression levels in treated samples were assessed as gene expression relative to housekeeping gene expression and relative to the control condition according to the − ΔΔCt method. For each data point, three independent samples were used, and each sample was run in duplicate. The following primers were used: human GAPDH (forward: TTGCCATCAATGACCCCTTCA, reverse: CGCCCCACTTGATTTTGGA), human indoleamine 2,3-dioxygenase (forward: ACCACAAGTCACAGCGCC, reverse: CCCAGCAGGACGTCAAAG) and human kynurenine 3-monooxygenase (forward: GATGAGGAAGATAAGCTGAGGC, reverse: CTTAAGGTTTCTTCCCCCTCTC). To ensure specificity of the amplified PCR products, for each run a post-amplification melting curve analysis was performed using the second derivate maximum method.

### HTRF cytokine measurements

For measurement of intracellular protein expression, supernatant was removed and cells were lysed in HTRF lysis buffer (PBS + 0.5% Triton-X). TNF-α and IL-6 levels were assessed using the respective HTRF assays (Cisbio Bioassays, Codolet, France), which were performed according to manufacturer’s instructions. Assay fluorescence was read on the Clariostar™ HTRF-compatible fluorescence reader (Clariostar, BMG Labtech, Ortenberg, Germany). Cytokine expression levels were normalized to total protein content of each sample. Total protein concentrations were assessed via BCA-Assay.

### Statistical analyses

Statistical analyses were performed using the statistical software GraphPad Prism (Ver. 5.04, GraphPad Software, La Jolla, USA). Differences between group means were analyzed via one-way ANOVA followed by Tukey’s post hoc test where appropriate. Values are presented as means ± standard deviations. *P* values < 0.05 were considered as statistically significant.

## Results

### Minocycline inhibits RA degradation in human monocytes

**Fig. 1:**
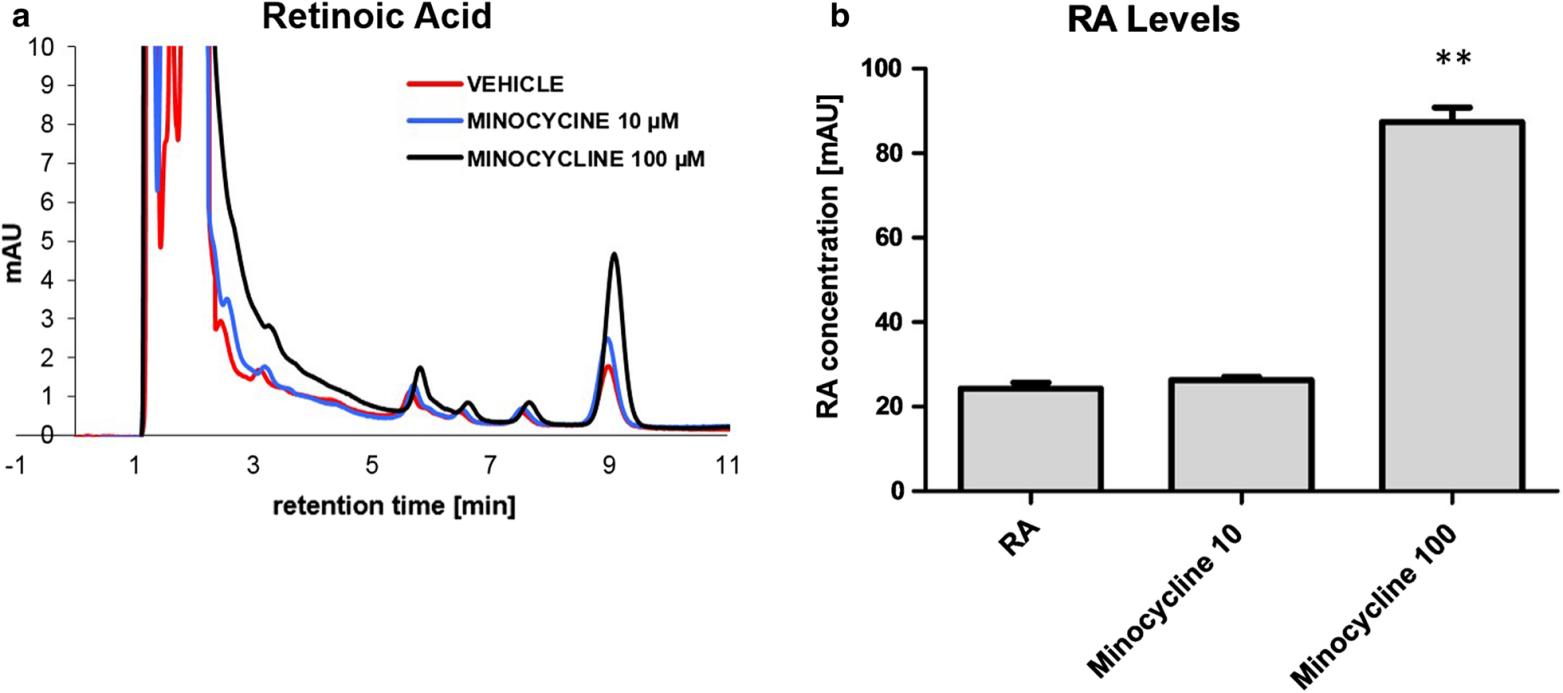
Minocycline inhibits RA-degradation in human monocytes. **a** Representative chromatographs of retinoic acid (RA)-measurements in cell culture supernatants from THP1-cells, each chromatograph representing only a single experiment. Degradation of RA is substantially inhibited in the presence of minocycline (MINO). All-trans-RA elutes with a retention time of ~ 9 min. **b** Quantitative analysis revealing an inhibition of RA-degradation in monocyte cells. While pretreatment with 10 µM minocycline reveals nearly no effect on RA levels, treatment with 100 µM minocycline results in a striking inhibition of RA-degradation. Results are presented as mean ± SEM, assessed in 4 independent experiments

Exposure of human THP-1 monocytes to the tetracycline antibiotic minocycline results in a substantial inhibition of the degradation of RA. RA was added to a final concentration of 1 µM subsequent to a 24 h treatment period with minocycline. After an additional 24 h incubation, RA levels and primary RA oxidation products (4-oxo- and 4-hydroxy-RA) were measured by HPLC. Vehicle-treated monocyte cultures exhibited a high rate of basal degradation of RA, as evidenced by decreasing RA levels and increasing levels of oxidation products. Treatment with minocycline effectively blocked this process in a concentration dependent manner (Fig. 1).

**Fig. 2:**
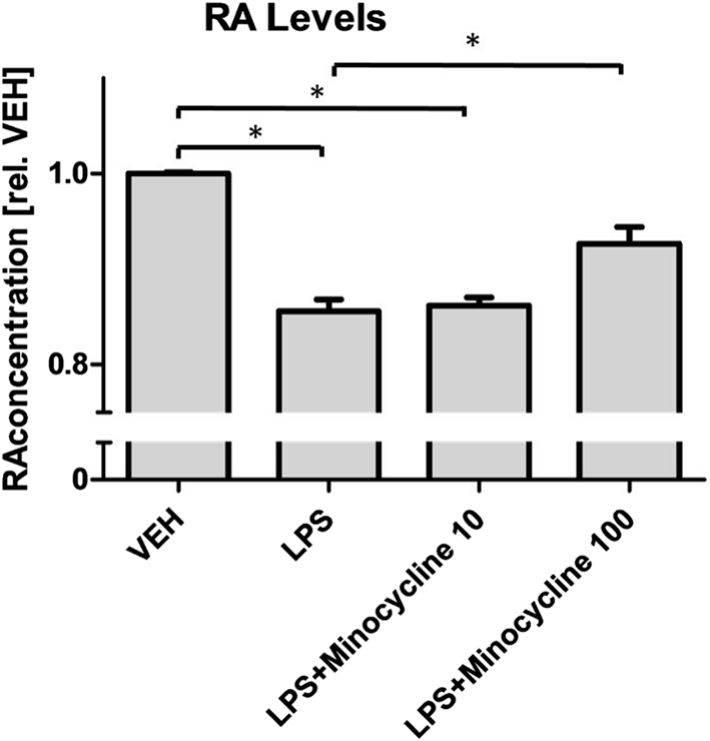
Minocycline prevents LPS-induced increase of RA-degradation in activated macrophages. LPS-stimulation significantly reduces retinoic acid (RA)-levels in THP1-cells. This effect can be reduced via minocycline: While pretreatment with 10 µM minocycline (MINO) results in a trend towards attenuating RA degradation, treatment with 100 µM minocycline results in a significant inhibition of LPS-induced RA-degradation. Results are presented as mean ± SEM, assessed in 3 independent experiments

Following up on own previous findings in murine microglia on inflammation-induced increase in RA-turnover [39], we sought to assess the same in human monocytic cells and to investigate the effects of minocycline on inflammation-induced RA-turnover. Minocycline- or vehicle-pretreated human monocytic cells were exposed to vehicle or LPS and allowed to degrade RA (1 µM) over a period of 24 h. RA levels were determined in cell culture supernatants after the incubation period, revealing a strong, concentration-dependent effect of minocycline in blocking the LPS-induced increase in RA turnover (Fig. 2).

### Minocycline-induced inhibition of IDO expression is retinoid-dependent

In order to assess a role for retinoid signaling in minocycline’s anti-inflammatory effects, we next examined the effects of minocycline on LPS-induced activation in the presence and absence of retinoids. We chose to quantify the expression of indoleamine-dioxygenase (IDO) and Kynurenine 3-monooxygenase (KMO) as relevant inflammation-associated enzymes that additionally play a key role in the kynurenine pathway, a process potentially related to the neuropsychiatric aftermath of chronically activated microglial cells.

**Fig. 3:**
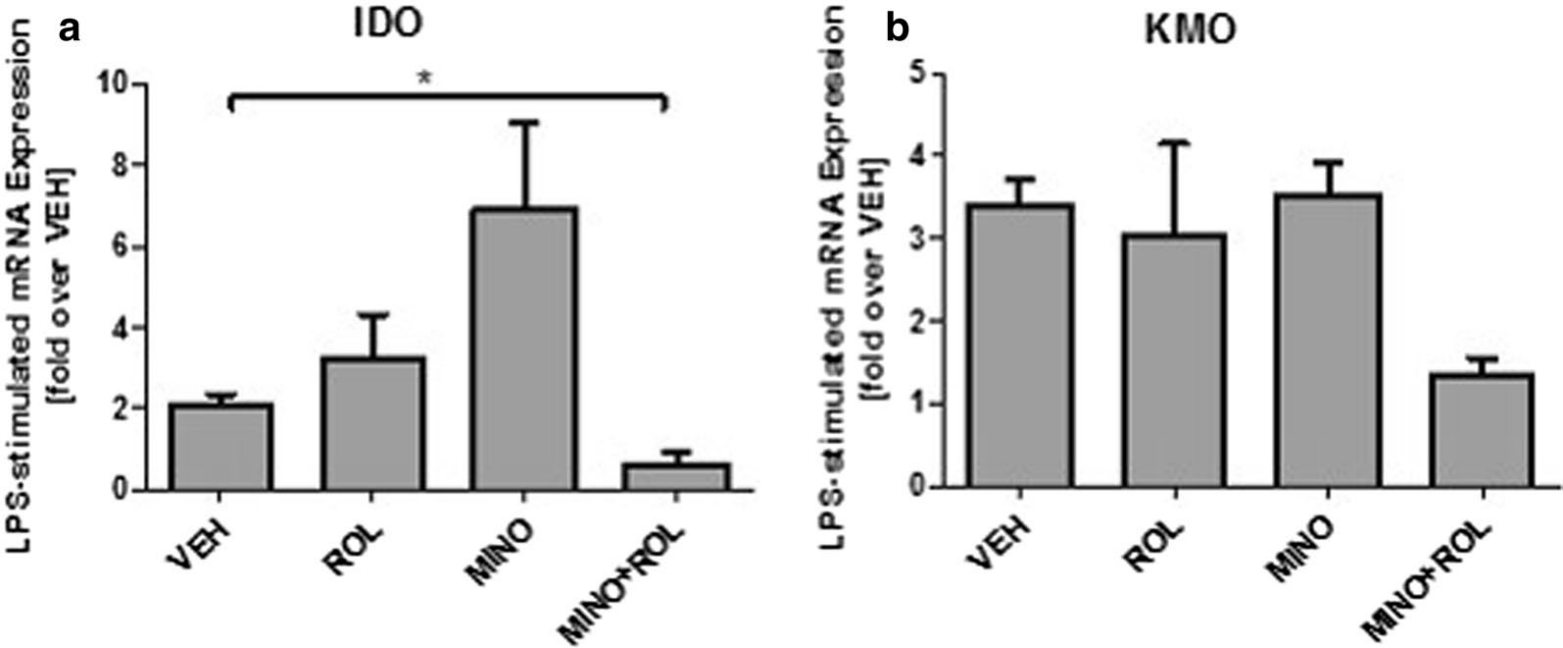
Expression of IDO and KMO after LPS-induced pro-inflammatory stimulation of human microglial-like cells. LPS induces an increase of the expression of the kynurenine pathway-enzymes IDO (**a**) and KMO (**b**). Minocycline (MINO) potently inhibits stimulation of indoleamine-2,3-dioxygenase (IDO) expression only in the presence of retinol (ROL) (**a**). For Kynurenine-3-monooxygenase (KMO), the same trend can be observed, however reaching no statistical significance (**b**). Results are given as fold over vehicle (VEH), mRNA expression was calculated relative to GAPDH housekeeping gene expression (ΔΔCT-Method). Results are presented as means +/- SEM from 4 healthy controls, assessed in 4 independent experiments

As demonstrated in Fig. 3, LPS-induced activation of human monocytic, microglial-like cells results in a robust increase of kynurenine pathway-related enzymes IDO and KMO, with a statistically significant increase for IDO and a trend towards increased expression for KMO (Fig. 3). Interestingly, pretreatment with both, retinol and minocycline alone resulted in an overall increased IDO and KMO expression with a stronger increase for minocycline, an effect that was more pronounced for IDO than for KMO expression. The combined treatment with minocycline and retinol, however, resulted in a striking, statistically significant decrease in IDO, and a slight trend towards reduced expression also for the KMO.

### Minocycline-induced inhibition of proinflammatory cytokines is retinoid-dependent

To assess whether the retinoid-dependent effects of minocycline on IDO expression may extend to more general, and functionally more relevant pro-inflammatory processes, we next studied the protein expression of the proinflammatory cytokines TNF-α and IL-6 using the same cell culture model based on human primary microglial-like monocytes.

**Fig. 4:**
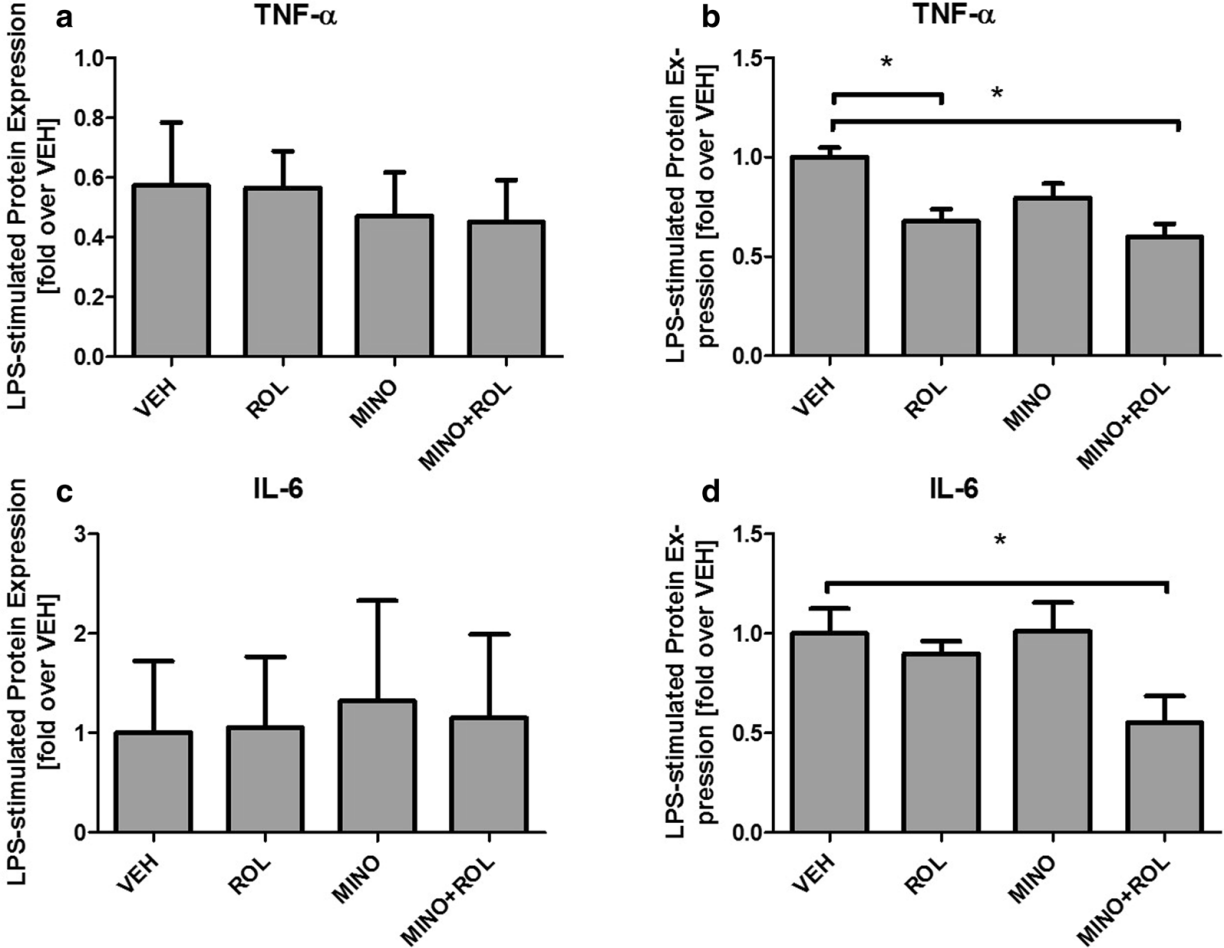
TNF-α and IL-6 synthesis after LPS-induced pro-inflammatory stimulation of human microglial-like cells. LPS-stimulated cytokine expression of human microglial-like cells: Minocycline inhibits the synthesis of pro-inflammatory cytokines TNF-α (**a**, **b**) and IL-6 (**c**, **d**) only in combination with retionids. Cytokine levels are presented relative to protein concentration. Tested conditions included vehicle (VEH), retinol (ROL), minocycline (MINO) and a combination of ROL and MINO, given as differences between LPS-stimulated and the unstimulated conditions. Values are given as mean ± SEM from at least four healthy controls in independent experiments

As expected, LPS treatment resulted in significantly increased cytokine levels of both, TNF-α and IL-6. Surprisingly, however, no inhibitory effect of minocycline, neither with nor without retinoids was observed (Fig. 4a, c). Based on the hypothesis that minocycline may promote its medium- to long-term anti-inflammatory actions indirectly via attenuating the homeostatic process of constant degradation of intracellular RA, we systematically added metabolically competent synaptosomal preparations from rat brain as a natural source of RA-degrading enzymes to cell culture media.

After the supplementation of cell culture media with synaptosomal preparations, co-treatment with minocycline and retinol again resulted in decreased TNF-α and IL-6 levels, while minocycline alone exhibited no influence on cytokine expression (Fig. 4b, d). Treatment with retinol alone resulted in a significant decrease of TNF-α expression (Fig. 4b). For IL-6 expression, without reaching statistical significance, the same trend was observed (Fig. 4d).

### Minocycline’s anti-inflammatory effects are mediated through RAR signaling

In order to identify the receptors involved in contributing to the anti-inflammatory effects of minocycline, we used a pharmacological approach based on the two well-established inhibitors of the two major retinoid receptors, the retinoic acid receptors (RAR) and the retinoid-X-receptors (RXR).

**Fig. 5:**
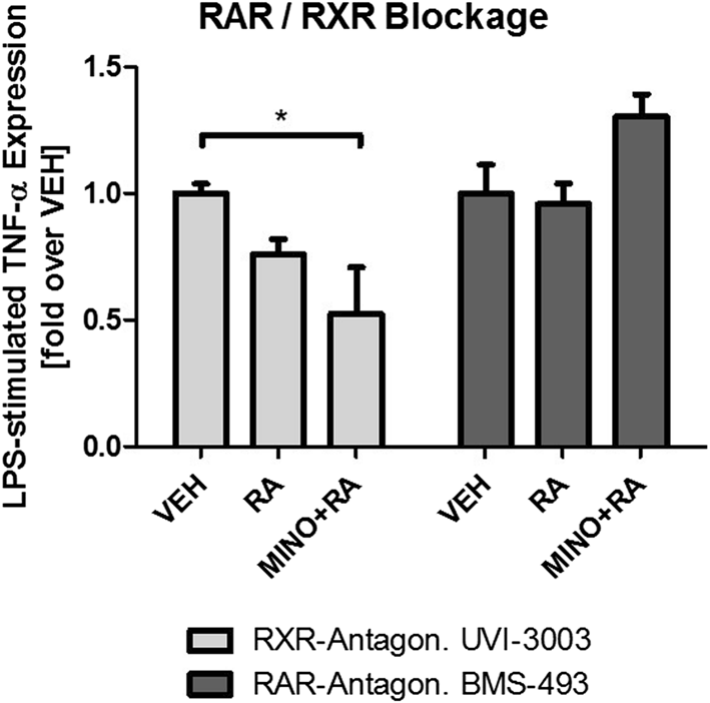
Pharmacological blockage of RA receptors RXR and RAR. Co-treatment with pan-retinoid-X-receptor (RXR) antagonist UVI-3003 (UVI) does not affect the anti-inflammatory effect of the combination of retinoic acid (RA) and minocycline (MINO) on TNF-α synthesis while pan-retinoic acid receptor (RAR) antagonist BMS-493 (BMS) completely impairs this anti-inflammatory mechanism. Results are presented as LPS-stimulated TNF-α, normalized for protein concentration. Values are given as mean ± SEM from at least four healthy controls in independent experiments

Using TNF-α protein levels as primary outcome parameter, human microglial-like cells were again treated with minocycline, retinoids and LPS as before, but prior to the addition of minocycline/retinoids, cells were treated either with the pan-RXR antagonist UVI-3003 (UVI) or the pan-RAR antagonist BMS-493 (BMS). Interestingly, pre-treatment with UVI remained without effect on the anti-inflammatory actions of minocycline + retinoids, and basically led to the same results that were seen in absence of the antagonist. Conversely, treatment with the pan-RAR antagonist BMS completely blocked the inhibitory effect of minocycline and RA (Fig. 5).

## Discussion

The potential contribution of microglia-driven neuro-inflammation to the pathogenesis of neuropsychiatric disorders gains more and more attention, and so are strategies that are aimed to specifically target this process. While microglial activation has been reported in a plethora of neuropsychiatric disorders, is appears to specifically play a role in those disorders that have been demonstrated to be associated, and hypothesised to be triggered by, chronic inflammatory processes. This has mainly been shown for primarily neurodegenerative Alzheimer’s disease, but also for major depressive disorder (MDD) and for negative symptoms in schizophrenia [23–32]. In MDD, an inflammatory pathogenesis is specifically hypothesized for those subjects that appear to be “treatment refractory” [17], so that novel strategies are warranted for both, diagnostic and therapeutic approaches.

Interestingly minocycline, a well-established tetracycline antibiotic, is also known from preclinical studies in rodents to act as a potent inhibitor of (chronic) microglial activation. Minocycline still enjoys great popularity in anti-acne therapy, likely for reasons of pleiotropic, anti-inflammatory actions [33], and also in other indications such as rheumatoid arthritis, minocycline treatment is discussed as an alternative treatment strategy in certain clinically severe cases [40].

With respect to possibly microglia-mediated neuropsychiatric conditions such as negative symptoms in schizophrenia, there is first evidence from controlled clinical trials, suggesting efficacy of minocycline [41] and our own clinical trial on the efficacy of minocycline in otherwise “treatment-resistant major depression” is currently ongoing (Clintrials.org ID: NCT02456948). We have previously demonstrated a link between minocycline’s mode of action and retinoid signaling [33, 34, 42].

While minocycline can readily block the degradation of RA in murine skin and brain tissue [33, 34], this mechanism has never been studied in human tissue before, and, more importantly, the suggestive evidence pointing towards a role for this mechanism in minocycline’s anti-inflammatory actions has not been investigated before. Additionally, most research on minocycline’s anti-inflammatory mechanisms has been conducted in non-primate cell systems.

RA is a CNS morphogen that is not only crucial for brain development, but also known to inhibit microglial activation [39] and neuro-inflammation [35, 43]. RA is a potent neuroprotective agent and known to play a key role in synaptic scaling [44]. Synaptic scaling is one of the major synaptic plasticity-associated mechanisms in the adult brain. In sum, all of these mechanisms are known to be affected in neuropsychiatric disorders. Moreover, there is a large body of direct evidence for an involvement of retinoid signaling in the pathogenesis of affective [45] and of other neuropsychiatric disorders [46], suggesting that local brain RA may function as an “endogenous antidepressant”. Furthermore, the catabolism of RA [47] is inhibited by the widely-used and well-established antidepressant fluoxetine, suggesting that fluoxetine’s neuroprotective, its anti-inflammatory—and potentially its anti-depressant properties may all together be mediated through RA signaling [48].

For the very first time, our data show a significant inhibition of RA degradation by minocycline in human monocytic THP-1 cells. Own previous data revealed the same effect of minocycline in human SH-SY5Y neuroblastoma cells [33, 34], underlining the hypothesis that neuroprotective and anti-inflammatory RA may be the “effector” for minocycline’s pleiotropic anti-inflammatory actions. Furthermore, our results reveal that inflammatory stimulation with LPS leads to an enhanced RA-degradation and consequently lower RA-levels in monocytes. Based on previous findings in other tissues such as murine microglial cells, this mechanism was not completely unexpected [39]. Finding the same effects in human cells, however, suggests that degradation of local RA may be a significant contributor to a pro-inflammatory microenvironment. With our demonstration of minocycline significantly blocking the inflammation-enhanced RA catabolism, we have identified a mechanism that might underlie minocycline’s pleiotropic effects, namely its inhibition of monocytic/microglial activation.

Subsequent experiments were thus designed to functionally assess an involvement of retinoid signaling in minocycline’s anti-inflammatory mechanisms in the human cell culture system. Pro-inflammatory conditions were simulated by using the established stimulation of cell cultures with LPS. As a readout, we chose key pro-inflammatory cytokines and enzymes of the kynurenine-pathway, a pathway that may be considered a central, inflammation-responsive pathway in activated brain macrophages. As a cellular model, we used primary human monocyte-derived macrophages that were differentiated using a set of selected cytokines, into more mature macrophages closely resembling the differentiation state of microglia. While most microglial cells in the adult CNS are thought to be derived from primitive yolk sac macrophages that have entered the brain during the embryologic period [49], microglia-like cells are also recruited from circulating monocytes [50] and continue to express numerous macrophagal receptors [51]. This makes patient specific macrophages an appealing model for studying the various aspects of monocytic/microglial cells that are claimed to be associated with psychiatric disorders. The use of peripheral monocytic cells and subsequent differentiation exhibits the advantage of easy accessibility and full genetic as well as epigenetic background of the patient. Nevertheless, it has to be pointed out that this is an in vitro model. Even though many physiological processes can be studied at a patient-specific level in vitro, there are numerous limitations to in vitro versus in vivo studies. A specific limitation concerns the cell line that was used to carry out the retinoid metabolism assays. This cell line certainly differs from the primary cells that were used for studying the inflammatory responses. Retinoid metabolism assays, however, only require intact human enzymes, for which the selected cell line represents an excellent source.

Our data reveal that LPS robustly induces the expression of IDO and KMO, the key enzymes in the kynurenine pathway, as well as inflammatory cytokine levels of TNF-α and IL-6, processes that have been associated with the pathogenesis of various neuro-inflammatory processes [7], and that have been linked to the pathogenesis of several neuropsychiatric diseases. TNF-α is known to be increased in anxiety disorder [52], posttraumatic stress disorder [53, 54] and major depression, where TNF-α and IL-6 are also associated with a smaller chance to respond to treatment with SSRIs [55]. Treatment with LPS, on the other hand, results in sickness behaviour that in many aspects resembles depressive symptoms [56], again pointing towards the relevance of these neuro-inflammatory processes. Pharmacological blockage of RAR/RXR retinoid receptors revealed that minocycline’s anti-inflammatory effects are likely mediated via RAR-receptors, since blockage of RXR receptors remained without effect, blockage of RAR-receptors however completely impaired minocycline’s anti-inflammatory effects as assessed by measuring TNF-α expression after LPS-stimulation.

The end product of microglial kynurenine pathway, quinolinic acid, is not only known to be a neurotoxic NMDA-receptor agonist [22] but furthermore to be elevated in the cerebrospinal fluid of suicide victims [57]. Cytokines [58] and LPS [59, 60] are known to induce key enzymes of the kynurenine pathway, resulting in increased quinolinic acid levels. Based on these findings, microglial activation is an interesting target for future treatment strategies in neuropsychiatric diseases [49] and minocycline is one promising substance to target this mechanism [61]. Nevertheless, despite the promising results of minocycline to inhibit microglial activation in in vivo models [1–6], our results reveal that minocycline alone does not inhibit LPS-stimulated increased IDO and KMO expression and cytokine levels, but requires the presence of retinoids to exert its anti-inflammatory actions. Furthermore, inhibitory effects of retinoids and minocycline were dependent on the presence of synaptosomal preparations in the assay. Based on previous findings of minocycline affecting RA metabolism [33–35], the added synaptosomal preparations represented the source of RA metabolism in the assay. This way we were able to precisely control for retinoid metabolism, indirectly demonstrating that the magnitude of minocycline’s anti-inflammatory effects was indeed dependent on the presence of RA metabolic enzymes.

## Conclusions

Taken together, our data suggest that retinoids may be required for minocycline’s anti-inflammatory effects on human microglial-like cells in vitro. Furthermore, our data may point towards an inhibition of local RA turnover and consequently increased levels of anti-inflammatory RA as the underlying mechanism. The RA-dependent mode of action for minocycline appears to be predominantly mediated through RAR-signaling.

## Abbreviations

BMS: BMS-493
DMEM: Dulbecco’s modified Eagle’s medium
FCS: fetal calf serum
IDO: indoleamine-2,3-dioxygenase
KMO: kynurenine 3-monooxygenase
LPS: lipopolysaccharide
MDD: major depressive disorder
PBMC: peripheral blood mononuclear cells
PTC: pseudotumor cerebri
RA: retinoic acid
RAR: retinoic acid receptor
RXR: retinoid-X-receptor
UVI: UVI-3003
